# Fine Mapping the Spatial Distribution and Concentration of Unlabeled Drugs within Tissue Micro-Compartments Using Imaging Mass Spectrometry

**DOI:** 10.1371/journal.pone.0011411

**Published:** 2010-07-14

**Authors:** Anna Nilsson, Thomas E. Fehniger, Lena Gustavsson, Malin Andersson, Kerstin Kenne, György Marko-Varga, Per E. Andrén

**Affiliations:** 1 Medical Mass Spectrometry, Department of Pharmaceutical Biosciences, Uppsala University, Uppsala, Sweden; 2 AstraZeneca R&D, Lund, Sweden; 3 Institute of Clinical Medicine, Tallinn University of Technology, Tallinn, Estonia; 4 AstraZeneca R&D, Södertälje, Sweden; 5 Clinical Protein Science and Imagining, Department of Measurement Technology and Industrial Electrical Engineering, Lund University, Lund, Sweden; University of Giessen Lung Center, Germany

## Abstract

Readouts that define the physiological distributions of drugs in tissues are an unmet challenge and at best imprecise, but are needed in order to understand both the pharmacokinetic and pharmacodynamic properties associated with efficacy. Here we demonstrate that it is feasible to follow the in vivo transport of unlabeled drugs within specific organ and tissue compartments on a platform that applies MALDI imaging mass spectrometry to tissue sections characterized with high definition histology. We have tracked and quantified the distribution of an inhaled reference compound, tiotropium, within the lungs of dosed rats, using systematic point by point MS and MS/MS sampling at 200 µm intervals. By comparing drug ion distribution patterns in adjacent tissue sections, we observed that within 15 min following exposure, tiotropium parent MS ions (mass-to-charge; m/z 392.1) and fragmented daughter MS/MS ions (m/z 170.1 and 152.1) were dispersed in a concentration gradient (80 fmol-5 pmol) away from the central airways into the lung parenchyma and pleura. These drug levels agreed well with amounts detected in lung compartments by chemical extraction. Moreover, the simultaneous global definition of molecular ion signatures localized within 2-D tissue space provides accurate assignment of ion identities within histological landmarks, providing context to dynamic biological processes occurring at sites of drug presence. Our results highlight an important emerging technology allowing specific high resolution identification of unlabeled drugs at sites of in vivo uptake and retention.

## Introduction

During the last decade, significant technological improvements in mass spectrometry have had a great impact on drug discovery and development [1], [2]. The direct analysis of tissue sections using matrix-assisted laser desorption ionization (MALDI) imaging mass spectrometry (IMS) is an emerging technology based on a surface sampling process. The technology allows analysis and visualization of endogenous proteins, peptides and lipids as well as exogenous molecular species, such as administered compounds, in their native biochemical states within the same tissue section with high molecular specificity [3], [4], [5], [6]. Molecular images are created by rasterizing over the sample while collecting MS or tandem MS (MS/MS) spectra from every position at a chosen resolution. The localization pattern from individual molecular species present on the tissue surface can then be extracted and positioned on the original histological image with the abundances represented by a concentration dependent color scale.

In the process of drug development for therapy purposes, one of the key objectives is to optimize the retention of a compound. There is however a limitation in discovery projects today where tissue retention is measured by analyzing the total concentration of drug in tissue homogenates extracted at different time points. These measurements cannot detect the differential distributions or relative differences in local concentrations of drugs within specific tissue compartments and may therefore not reveal the correct information on why a compound with high retention does not always provide long effect duration.

Several recent studies have reported MALDI IMS techniques to localize small molecular mass pharmaceutical compounds in tissue [5], [6], [7], [8], [9]. These reports include applying MALDI IMS to whole body sections of animals dosed with drugs by oral or intravenous routes and have demonstrated the potential of differentially measuring drug/metabolite ion signatures in separate organ compartments [10], [11], [12].

In this study we provide the first evidence that compounds administered by inhaled delivery at standard pharmacological dosage can be quantitatively detected by MALDI IMS with accuracy and precision. We have used tiotropium, a bronchodilator used in the management of chronic obstructive pulmonary disease as a model drug. MALDI IMS is a technology application that requires no labeling of the native compound structure that could potentially change the properties of the drug. We demonstrate that using a conventional MALDI IMS platform, and dedicated MALDI data processing tools, we can congruently overlay the array of drug distribution with exact histological context within specific tissue compartments at 200 µm increments. Our study demonstrates that a combined approach of MALDI IMS and high resolution histology can be effectively applied to quantitatively and qualitatively determine in vivo drug uptake into targeted tissues.

## Results

### Detection and distribution of tiotropium in tissue sections by MALDI IMS

We have used a rodent model to study drug distribution in the lung using a respiratory inhalation model with the reference compound tiotropium bromide (TTP), a prescription muscarinic antagonist that affects bronchodilation. TTP (systematic International Union of Pure and Applied Chemistry, IUPAC, name ((1α,2β,4β,7β)-7-[(hydroxidi-2-thienylacetyl) oxy]-9,9-dimethyl-3-oxa-9-azoniatricyclo[3.3.1.0^2,4^]nonane) is administered as an inhaled bronchodilator for the treatment of asthma and chronic obstructive pulmonary disease (COPD).

**Figure 1:**
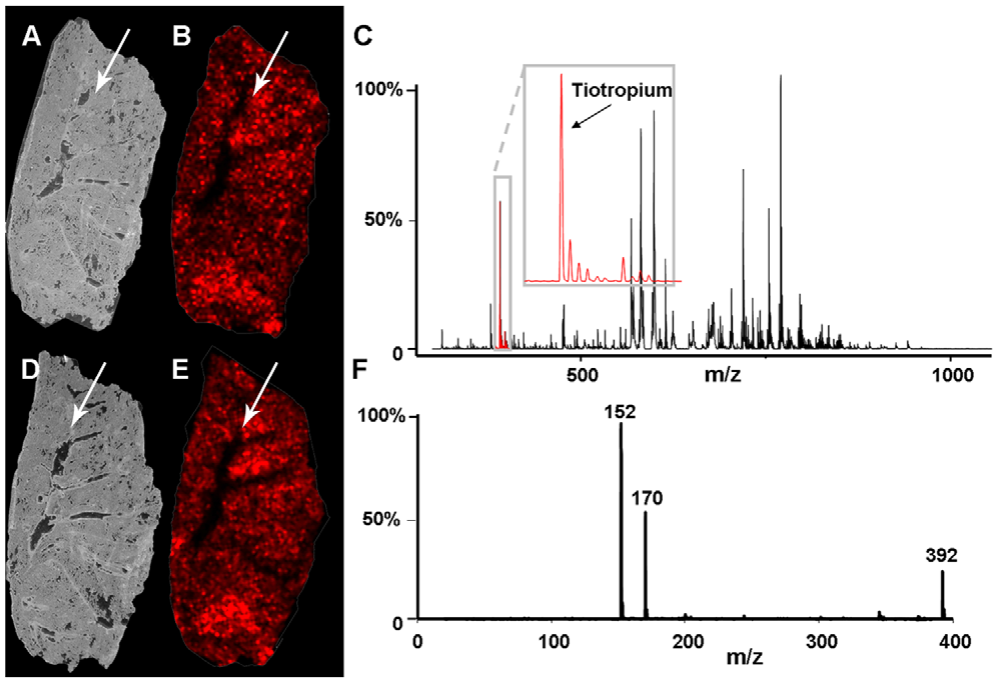
The distribution of tiotropium on two central lung tissue sections from a rat after inhalation dosing. The arrows indicate the route of drug delivery. (A) Photographic image of a rat lung tissue section analyzed by MALDI IMS with (B) the obtained distribution of tiotropium represented by the parent ion m/z 392.1. (C) A typical MS spectra obtained by MALDI MS analysis on rat lung tissue. (D) Photographic image of a rat lung tissue section analyzed by MALDI IMS/MS with (E) the obtained distribution of tiotropium represented by the daughter ion m/z 152.1. (F) MS/MS spectra of tiotropium. The distribution of tiotropium obtained in MS mode (B) correlate very well with the distribution pattern obtained by rastering over the tissue in MS/MS mode (E). The MALDI matrix CHCA was applied by an automatic sprayer device.

Initially, both MS and MS/MS methods were used to identify and then validate the detection of a tiotropium chemical standard both on MALDI target plates and embedded by spotting onto the tissue matrix. Structural information and mass identity of TTP, obtained from the drug standard, showed a characteristic MS signal (m/z = 392.1) and MS/MS fragments at m/z 152.1 and 170.1. We administered TTP to rats for fifteen minutes in a two-stage flow-past inhalation chamber, as an aerosol of a nebulized solution, at a dose of 1.1 mg/kg (total delivered lung dose of 50 µg). Serially sectioned slices of lung were dried onto conductive MALDI plates for MS analysis or for histological analysis. Careful preparation allowed preservation of the histological compartments of the conducting airway tree absorbing the inhaled TTP in individual thin sections (Fig. 1 A, D). The TTP distributions in lung tissues from dosed animals were analyzed by MALDI IMS at a resolution of 200 µm in both MS and MS/MS mode (Fig. 1). The MALDI IMS images showed that TTP was rapidly transported from the central conducting airways to alveolar beds in the upper and lower parenchyma. Spectra from lung tissue of dosed animals displayed matching daughter ions (m/z 152.1 and 170.1) confirming that TTP was indeed detected in lungs of dosed animals. In MS mode, each pixel was normalized against its total ion current [13], [14]. After normalization of data in MS mode, the distribution of TTP correlated well with the distribution pattern obtained by MS/MS imaging on a consecutive section.

### Tiotropium concentration in lung tissue sections

**Figure 2:**
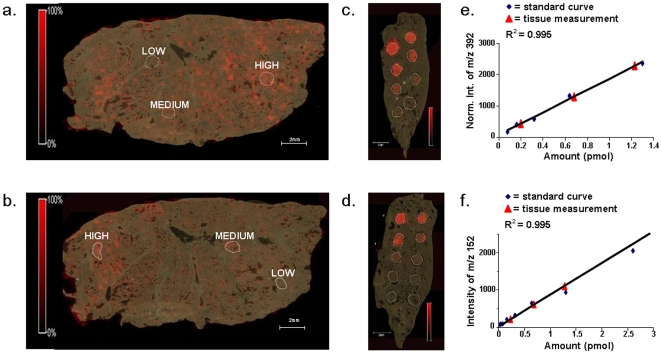
Estimation of tiotropium levels in lung tissue from rats dosed with the drug (a, b) by comparison to drug standard samples spotted on control tissue (c, d). Control lung tissue was spotted with different amounts of tiotropium. In total, 20 fmol*, 40 fmol*, 80 fmol, 160 fmol, 320 fmol, 640 fmol, 1.3 pmol, 2.6 pmol, 5 pmol, or 10 pmol were placed on the control tissue (c, d). The spotted controls were coated with matrix using an automatic spraying device and analyzed on the same MALDI target as the tissue sections from the animals dosed with tiotropium. One pair of samples (spotted control and dosed tissue) was analyzed in MS mode (a) and one pair was analyzed in MS/MS mode (b). Average intensities from the tiotropium spiked regions were calculated and a standard curve was constructed from these values for the MS and MS/MS experiments, respectively (e, f). Three different regions, representing low, medium and high intensity areas, respectively, were selected on the dosed tissue sections (a, b) and the average intensities of these were matched to the standard curves. The color intensity scales on the controls (c, d) were set to reflect 0–100% of the maximal peak intensity of the sections from the dosed animals. Hence, this saturated the pixels on the controls with a higher intensity than the maxima of the dosed tissues. Calculated tiotropium levels from the high, medium, and low intensity regions on the dosed lung tissue sections (f). The levels obtained in MS mode correlate very well with the levels measured by MS/MS. (*only in MS/MS mode).

We quantified the regional levels of TTP in lung tissue by simultaneous MALDI IMS analysis of lung tissue from dosed animals and serial dilutions of the drug deposited on control lung tissue. Two lung tissue sections, one control and one from dosed rats, were placed on the same MALDI target slide. Different concentrations (20 fmol-10 pmol) of drug standards were applied to the control tissue and both sections were coated with matrix at the same time in the automated spraying device. Two sets of pairs were analyzed by imaging MALDI MS and MS/MS mode, respectively, at a spatial resolution of 200 µm. After analysis, the average intensity values from each TTP deposit were calculated and a linear regression concentration curve was constructed for the MS and MS/MS experiment, respectively (Fig. 2). There was a slight delocalization of TTP ion outside the spotted area for the highest concentration of drug (Fig. 2). Apart from this, there was no TTP signal on the non-spotted area of the scanned control tissue. In MS mode there was linearity between 80 fmol to 2.6 pmol (r = 0.983) and in MS/MS mode linearity was obtained in the range from 80 fmol to 5 pmol (r = 0.995).

Spectra from three regions of interest on the tissues from dosed animals representing high, medium, or low intensities were extracted (Fig. 2). The average intensities of the pixels in the regions were calculated after spectra processing and normalization (MS mode). The obtained values were recalculated to TTP amounts using the standard curves (Fig. 2). The drug standard curve obtained from the pre-spotted tissue proved to be linear in the range corresponding to levels obtained on the tissue isolated from rats administered with the drug. The levels of TTP in low, medium and high intensity regions correlated very well between the MS and MS/MS experiments (Table 1). There was about a six times higher concentration of TTP in a high intensity region (∼1.3 pmol) than in a low intensity region (∼0.2 pmol).

**Table 1:** The amount of tiotropium in rat lung tissue from animals dosed with the drug analyzed using MS or MS/MS mode.

	High int. area	Medium int. area	Low int. area
MS	1.25 pmol	0.65 pmol	0.20 pmol
MS/MS	1.30 pmol	0.67 pmol	0.23 pmol

The levels presented as low, medium, and high regions correspond to the marked circled areas in Fig. 1 A, B (int.; intensity).

A conventional quantification technique i.e. homogenization of tissue, extraction of tiotropium, and analysis of tiotropium in lung tissue extracts using liquid chromatography (LC)-MS/MS including standard curves was applied to analyze the amount of tiotropium in lung tissue after inhalation dosing. The average concentration of tiotropium in whole lung tissue 15 minutes after completed inhalation was 8.9±3.0 nmol/g (pleural (∼7 nmol/g) and central (∼12 nmol/g)) corresponding to approximately 8% of the lung dose. This finding was consistent with a rapid absorption of tiotropium into blood after administration to the lungs of rats [15] and the levels correlated well with the concentrations of tiotropium obtained by MALDI IMS. For the first time, the regional ion intensities obtained from inhaled TTP could directly be translated to the distributed drug levels in the tissue sections.

**Figure 3:**
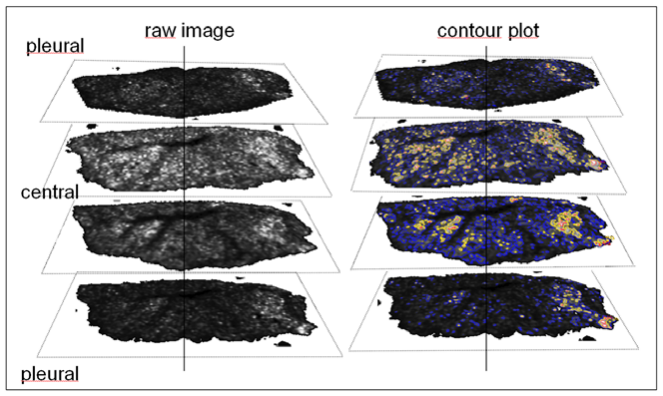
The spatial distribution of inhaled tiotropium in serially sectioned rat lung tissue. Raw MS images (left column) showing MS localization of inhaled tiotropium (*TTP*) ion (m/z 392) by pixel location in serial sections of whole lung moving anatomically in lung volume from pleural to central to pleural. The arrow indicates approximate carinal entry point of the drug into the central conducting airways. Contour plots (right) of the relative concentration gradients of *TPP* found in the various lung segments showed a rapid and homogeneous transport of the drug from airways into the parenchyma within 15 minutes after exposure. The MALDI matrix CHCA was applied by an automatic sprayer device.

To further investigate the regional dispersion pattern of the drug in the lung compartments, we compared sections of lung that represented serial samplings from the pleural surface to the central core of the lung volumes. As shown in Fig. 3, TTP was uniformly transported out of the central conducting airways into the small bronchioles and alveoli, and out to the pleural boundary. This is shown in the contiguous distribution and relative signal strength of TTP ions across the lung volume represented in individual lung slices.

Using open access software, the raw images of ion identities can be visualized at pixel coordinates corresponding to the original XYZ positions of MALDI IMS sampling. These images allow relative appraisals of concentration gradients for individual ion species throughout the tissue sample. However, these low resolution raw images are not adequate to allow detailed histological examination of the exact areas represented by the ion maps. In order to create an exact histological reference to these pixel locations, we re-plotted the raw ion maps by pixel location with colored contour plots based upon gray scale intensity (Fig. 3). We believe that the concentration scale revealed in these contour images provides a valid and unbiased estimation of the diffusion pattern of the drug in vivo relative to the route of delivery and the activity of drug transporters. This is new and valuable information that until now could not be obtained using conventional methodology such as extraction or autoradiography. In this case, the *TPP* distribution pattern is remarkable in the context of the homogeneous pan-alveolar uptake and the speed in which this occurred in vivo.

### Endogenous tissue ions: distributions related to histology

**Figure 4:**
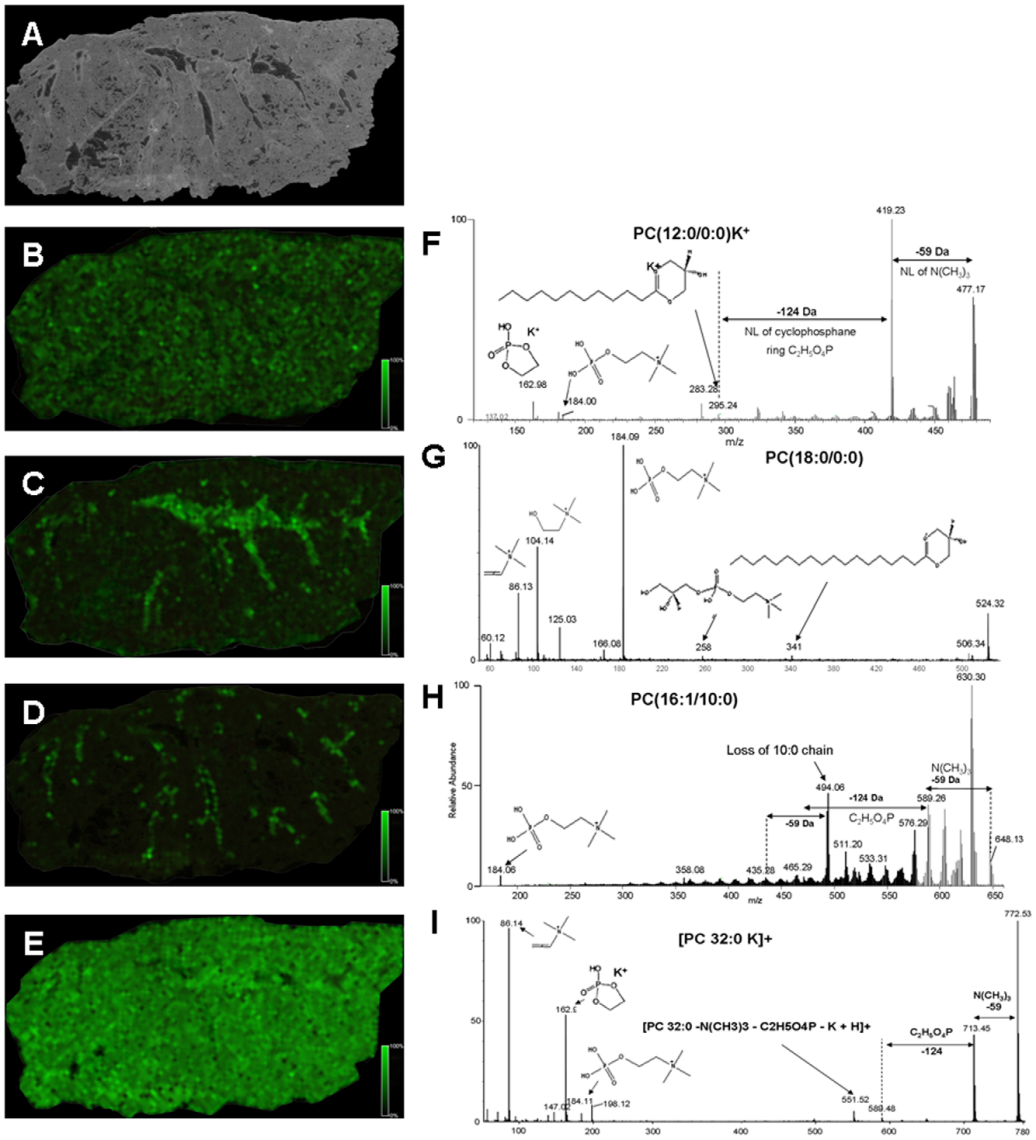
The distribution of endogenous molecules in lung tissue compartments by MS and identification by MS/MS. Photograph of rat lung tissue (A). Panel B–E displays the distribution of four different endogenous molecules present in (A). These molecules were identified as different phosphatidylcholines by MS/MS directly on the tissue section. (F) MS/MS spectrum of PC(12∶0/0∶0)K^+^, (G) MS/MS spectrum of PC(18∶0/0∶0), (H) MS/MS spectrum of PC(16∶1/10∶0), (I) MS/MS spectrum of PC(32∶0)K^+^. The MALDI matrix CHCA was applied by an automatic sprayer device.

Acquiring data directly on the tissue sections in MS mode gives the advantage of obtaining localization patterns of endogenous peptides, proteins, and phospholipids at each sampling location. We observed that ∼200 specific mass identities between 400–2200 m/z units could be localized on a single section using the given MALDI matrix optimized to detect the drug. Many of these endogenous molecules displayed a defined localization that correlated to specific tissue compartments (Fig. 4 C, D) while other compounds were more ubiquitously expressed throughout the tissue sample (Fig. 4 B, E).

**Figure 5:**
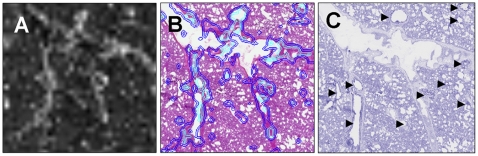
Localization of endogenous phosphatidylcholines (PC) ions in specific histological compartments. We detected a variety of PC ion species in the global MALDI IMS scans of lung tissue. Shown here is the distribution profile of PC m/z 674.6 detected as a raw image by pixel location (A) and as contours of the ion intensity (color code: aqua/high intensity, blue/medium intensity) that have been re-plotted onto scanned images of the original sample after histological processing and staining (B). PC m/z 674.6 was closely associated with the location of small bronchioles throughout the lung section. Deconvolution of the H&E stained image (C) allowed clearer visualization of these bronchioles (indicated by arrows) supporting this observation. The MALDI matrix CHCA was applied by an automatic sprayer device.

We submitted a variety of these ions to MS/MS analysis in an attempt to acquire a specific identification and identified multiple phosphatidylcholine (PC) molecules, such as PC(12∶0/0∶0)K^+^ m/z 478.2, PC (18∶0/0∶0) m/z 524.3, PC(16∶1/10∶0) m/z 648.5, and PC(32∶0) m/z 772.5 (Fig. 4 F–I, respectively). Characteristic fragments in the MS/MS analysis of PCs are neutral losses of trimethylamine [N(CH3)_3_], the cyclophosphane ring C_2_H_5_O_4_P, and the phosphocholine head group fragments (m/z 184, 125, 104, and 86). Potassiated or sodiated PCs, display characteristic fragments at m/z 163, and m/z 147, respectively [16], [17]. To further understand the anatomical distribution of these identified ions, we removed the matrix and histochemically stained the lung sections using conventional hematoxylin and eosin staining and scanned the slides using high definition digital scanning. We then matched and re-plotted the ion maps obtained with the original images of the tissue obtained on the MS instrument with the high definition images using software that created contour plots based upon gray scale intensity. We report here our initial observation that several of the PC ion species showed selective tissue compartment localization within the lung. For example, PC ion m/z 674.6 was tightly localized to areas surrounding small bronchioles. The intensity map of this ion (Fig. 5A) was subsequently processed into an intensity contour plot that was superimposed on the histological stained section (Fig. 5B). PC 674.6 was clearly and remarkably associated with bronchiole tissue (arrows in Fig. 5C), as seen in processed deconvolution image of the histology slide. MS/MS analysis of the ion m/z 647.6 confirmed the phosphocholine head group fragment but the exact tail composition of the ion could not be determined.

## Discussion

The present study is focused on the new emerging area of imaging the distributions of drugs in tissue compartments and presents new possibilities in an area of science that has remained dormant for decades. It presents an outline for surmounting a very difficult problem in modern pharmacological studies, that is, the direct quantitation of drug distributions within cellular environments. Using an experimental model that surveys sections of the whole lung lobe at a spatial resolution of 200 µm, we have applied MALDI IMS to identify and quantify the distribution patterns of a reference compound inhaled into the lung. This is the first report of the absolute quantification of an unlabeled drug within the individual components of a tissue.

The benefits of using MALDI IMS to detect administered drugs are several. First, the technique does not require exogenous labeling in order to trace the deposition of the drug. Any chemical modification of a small molecular weight compound could change the chemical and biological properties of the native structure. As such, the measurements by MALDI IMS differ from traditional molecular imaging of drugs by PET or autoradiography that require highly specific activity labels for detection and quantification. Second, the MALDI IMS has the advantage of delivering exact mass identifications with high confidence throughout the entire sample. One can literally follow the mass identities into any biological compartment that may or may not be related by biological function or structure to adjacent compartments. Third, MALDI IMS enables mapping of metabolic products or modifications of the compound at each site of MALDI IMS sampling, provided that the metabolites have sufficient ionization efficiency. Neither PET nor autoradiography can distinguish between a labeled parent and labeled metabolite of a drug at the site of measurement because it is the label rather than the drug which is being recorded. In addition to the drug, other ions representing proteins, peptides and lipids can be obtained at every sampling site which allows relative comparisons to important biological comparators present in each compartment. Last, the spatial resolution of MALDI IMS reported here (200 µm) is considerably better than the reported resolution values in whole tissue/organs by positron emission imaging (∼5 mm) [18] and magnetic resonance imaging (∼1 mm) [19]. Although MALDI IMS instruments currently on the market does not yet resolve at the single cell level in whole tissue, such developments in the MALDI IMS technology have been performed. Recent reports indicate technology advancements capable of irradiating and imaging areas with a resolution as small as <1 µm [20]. A MALDI IMS experiment on tissue sections has been carried out with a resolution of 7 µm in diameter [21]. These improvements would be of even more utility in studying drug efficacy.

## Methods

### Animals

The study was conducted in accordance with the European Communities Council Directive of 24 November 1986 (86/609/EEC) and was approved by the Lund/Malmö, Sweden Ethical Committee on Animal Experiments (no. M84-05). Male rats (n = 5, Wistar, Taconic, Denmark) of approximately 350 g body weight were used. The animals were allowed a 5 days acclimatization period prior to experimentation. They were kept in a climate controlled facility (22±3°C, 55±10% relative humidity) with 12 hours light-dark cycle. Food and water were given *ad libitum*.

### Drug administration and tissue collection

Tiotropium was administered as an aerosol of a nebulized solution to rats at a total dose of 1.1 mg/kg, resulting in a lung deposited dose of 50 µg. The rats were exposed to the aerosol in an in-house built two-stage nose-only flow-past inhalation chamber (Battelle Design) [22]. The lung dose was estimated from the aerosol concentration, measured by filter sampling at one port of the inhalation chamber. The amount of compound on the filter was analyzed by LC-MS/MS and the dose was calculated as previously described [22].The animals were anesthetized and sacrificed fifteen min after administration. The lungs were rapidly dissected out and immediately placed in a fulminating bath of isopentane/dry ice on hard plastic dish. The lungs were stored at −80°C until sectioned.

### Tissue sectioning

The frozen tissues were cut into 15–20 µm thick sections with a cryo-microtome (Leica CM3050S, Leica Microsystems, Germany). The lungs were cut along the long flat frontal plane of the left lobe from side to side to get full sections of the central airways. The sections were transferred to and thaw mounted on conductive MALDI glass slide dried and stored at −80°C until further analysis. Before matrix application, the tissues sections located on the MALDI glass slides (Bruker Daltonics, Bremen, Germany) were desiccated for 60 min under low vacuum at room temperature. In some experiments sections on MALDI slides were stained using conventional hematoxylin and eosin methodology after MS or MS/MS analysis.

### MALDI IMS matrix conditions

For manual spotting, α-cyano-4-hydroxycinnamic acid (CHCA) was prepared in 50∶50∶0.1 acetonitrile (ACN)/H_2_O/trifluoroacetic acid (TFA) under saturated conditions. The tiotropium drug standard (0.9 ng/µl) was mixed (1∶3) with matrix solution and 0.5 µl were placed onto a MALDI target (steel plate) or a control lung tissue for analysis. Matrix solution without tiotropium was placed on tissue sections as control to check for matrix derived peaks interfering with the mass of the drug.

For the imaging MALDI MS experiments the lung tissue sections were coated with CHCA matrix solution (5 mg/ml) in 50∶50∶0.1 ACN/H_2_O/TFA using an automatic spraying device (Image Prep, Bruker Daltonics). The Image Prep instrument deposits matrix solution onto the tissue in a controlled manner. Briefly, a matrix aerosol is created by vibrational vaporization under controlled conditions with all droplet diameters ≤50 µm and an average droplet size of ∼20 µm. A modified customized method was used for matrix application using the ImagePrep instrument. The parameters for incubation time, wetness and matrix thickness were optimized to get the best spectra possible without delocalization of drug or endogenous compounds on the tissue. The thickness of the matrix layer was monitored by the output from the optical sensor. In short, the method contained four identical phases in which a matrix layer corresponding to 0.3 V was added by repeating a spray cycle of 2.5 s followed by 10 s incubation time and 90 s of drying time (influx of N_2_). The glass slide was turned 180° in between each phase and the total matrix layer thickness added was 1.2 V output from the optical sensor. The coated tissue sections were dried in the desiccator for about 20 min before MS analysis.

### MALDI IMS detection limits and MS/MS verification

The range of detection was investigated by applying different concentrations of tiotropium onto a control lung section. The drug was mixed (1∶3) with matrix solution and the different amounts of tiotropium applied to the tissue were 10 pmol, 2.6 pmol, 640 fmol, 320 fmol, 160 fmol, 80 fmol, 40 fmol, and 20 fmol in a total volume of 0.5 µl. The matrix spots were analyzed in reflectron mode collecting 300 shots per spot. Using MS mode, tiotropium was detectable down to 80 fmol and the signal response was linear up to 2.6 pmol (R^2^ = 0.998).

The drug standard was analyzed in MS/MS mode using a LIFT method [23]. The LIFT method was calibrated on the CHCA matrix peak of 379.1 Da and its fragments. The same method was used to perform MS/MS directly on dosed lung tissue and spectra were compared to the MS/MS spectra generated from of the drug standard. Using MS/MS mode, tiotropium was detectable down to 40 fmol and the signal response was linear up to 10 pmol (R^2^ = 0.999).

### Direct analysis of tiotropium on tissue sections by MALDI MS and MS/MS

MS experiments were carried out using the UltraFlexII MALDI-TOF/TOF MS (Bruker Daltonics) equipped with a solid-state Smartbeam laser operating at 200 Hz. The MS spectra were acquired in positive reflectron mode. For the initial analyzes of the manually deposited spots, spectra consisting of 1000 laser shots were acquired in bundles of 5×200 shots and data was collected between m/z 300–2500 Da, unless stated differently. Prior to analysis the MS method was calibrated using a standard peptide mix (Bruker Daltonics) and the matrix cluster peak from CHCA at m/z 379.1 Da. The MS/MS experiments were performed using a LIFT method optimized for the drug by specific tuning of the timing of the LIFT cell and of the precursor ion selector.

The software Flex Imaging 2.0 (Bruker Daltonics) was used to set up the acquisition of the imaging experiments. The imaging MS experiments were performed by collecting spectra (200 or 300 shots) at a resolution of 175–200 µm in the same m/z range as above.

The spectra were baseline subtracted (Convex hull) and smoothed (Savitzski golay) in the processing software during acquisition. In MS mode, all spectra were normalized against total ion current to reduce influences by i.e. matrix hot spots [14], [24]. Here, we define the total ion current as the sum of all intensities in the mass range analyzed (300–2500 Da). For the sections analyzed in MS/MS mode, only fragment spectra were acquired from each spot and 400 laser shots were summed up in a random walk pattern from each position. The tiotropium fragments with m/z 152 Da and m/z 170 Da were used to visualize the distribution of tiotropium. The raw data from the imaging experiments were converted into BioMap format (Novartis, Basel, Switzerland), using an in-house written script and two dimensional ion density maps were created using FlexImaging (Bruker Daltonics) or BioMap.

### Estimation of tiotropium levels in tissue sections by imaging MALDI MS and MS/MS

One section of control lung tissue was placed on the same MALDI target as a central lung section from a dosed animal. Different amounts of tiotropium, 20 fmol-10 pmol (in 50∶50∶0.1 ACN∶H_2_O∶TFA) were spotted onto the control tissue in a total volume of 0.5 µl. The two sets of tissue sections (dosed animal and spotted control) were then coated with matrix as described above using the Image Prep and subsequently analyzed by imaging mass spectrometry in MS and MS/MS mode, respectively.

Average spectra were calculated from each of the tiotropium spotted regions on the control tissue and the linearity of the peak intensity (and peak area) of either the precursor ion or the fragment m/z 152 were calculated. Images were produced and the color intensity was adjusted so that the intensities on the spotted control matched the intensities on the dosed tissue. Average spectra were also calculated for three different areas on the dosed tissue sections. The areas were chosen as regions containing low, medium, or high intensity pixels and each area contained about 20–25 pixels. A linear regression curve was calculated from the spotted control and the levels of tiotropium on the dosed tissue were match against the standard curve. The absolute concentration of TTP on the dosed tissue sections was estimated by calculating the weight of the tissue of the selected tissue areas based on the volume of the tissue (mol/g).

### Quantification of tiotropium in lung tissue using LC-MS analysis

The concentration of tiotropium in lung tissue after inhalation dosing to rats was quantified after homogenization of a whole rat lung or lung tissue slices. The lung tissue was pulverized using a Covaris CryoPrep (Covaris Inc., Woburn, MA). Ringer buffer solution was added (3 mL buffer per g tissue) and the compound was extracted using adaptive focused acoustic ultrasound equipment (Covaris E210, Covaris Inc.). The samples were precipitated by addition of acetonitrile containing 1% acetic acid (1∶3 v∶v), centrifuged and the supernatant diluted with water (1∶4 v∶v). Samples were injected onto a LC system (Shimadzu LC-10ADvp pump and controller SCL-10Avp, Tokyo, Japan) equipped with a precolumn (Aquasil 5 µM 10×1 mm; Thermo Scientific, Stockholm, Sweden) and separation was performed on an analytical column (Aquasil 5 µm, 30×1 mm; Thermo Scientific) using a gradient of acetonitrile/water 99.5/0.5 (v/v) to acetonitrile acetonitrile/water/acetic acid 95/4.5/0.5 (v/v/v) as mobile phase at a flow rate of 0.25 mL/min (run time 4 min). Mass spectrometry was performed on a triple quadrupole mass spectrometer (Sciex API 3000, Concord, Ontario, Canada) with an electrospray ionization (ESI) source. ESI was performed in the positive ion mode with nitrogen as the nebulizer, heater and curtain gas. The detector was operated in the multiple-reaction monitoring mode using the transitions from the protonated molecules at m/z 392.1 to 152.1 for tiotropium. Budesonide was used as internal standard. Tiotropium standard solutions and the internal standard solution were prepared in acetonitrile.

### Identification of endogenous compounds by MS/MS directly on tissue sections

For the MALDI MS/MS experiments the lung tissue sections were coated with CHCA matrix solution (5 mg/ml) in 50∶50∶0.1 ACN/H_2_O/TFA using a glass spray nebulizer (TLC sprayer, Sigma Aldrich). At least 20 spray cycles were repeated manually at a 45 s interval to get a uniform coating. MS/MS analysis directly on tissue sections were performed using either a MALDI SYNAPT HDMS system (Waters, Manchester, UK) or a MALDI hybrid LTQ Orbitrap (Thermo Scientific, San Jose, CA). MS/MS spectra were collected from all over the tissue sample. The MALDI SYNAPT was operated in V-mode, m/z range of 50–1000 and the quadrupole transmission window was set to 1 Da for precursor ion selection. Using the MALDI Orbitrap ion isolation and collisional dissociation were achieved in the linear ion trap, and both precursor and product ions were detected in the Orbitrap. Full MS data were acquired at the 60,000 resolving power setting. Tandem MS data were acquired at the 7500 resolving power setting.
